# Periorbital squamous cell carcinoma with simultaneous adenocarcinoma in the right orbit

**DOI:** 10.1016/j.ijscr.2020.02.006

**Published:** 2020-02-06

**Authors:** Carmen Lok Tung Ho, Bhavin Visavadia, Keval Shah, Manjiri Deshmukh

**Affiliations:** a Imperial College School of Medicine, Imperial College London, Fulham Palace Rd, London W6 8RF, United Kingdom; b Northwick Park Hospital London, HA1 3UJ, United Kingdom

**Keywords:** Case report, Maxillofacial, Oncology, Adenocarcinoma

## Abstract

**Introduction:**

Cutaneous squamous cell carcinoma (cSCC) is an invasive and malignant tumour that is common in the orbital region. However, adenocarcinoma of the lacrimal gland has an incidence of less than one case per million per year. We present a patient who experienced a cSCC and adenocarcinoma of the lacrimal gland, which is unusual for both these cancers to present in the same patient in such close proximity.

**Presentation of case:**

A 47-year old male patient was referred for management of a cSCC of the right upper eyelid. This was treated surgically by means of wide local excision and orbital exenteration. Histological analysis of the resection specimen found an incidental synchronous lacrimal gland adenocarcinoma. To our knowledge this is the first case report to describe this phenomenon.

**Discussion:**

Although SCC is the most common secondary epithelial neoplasms of the orbit region, adenocarcinomas are rare in this same region. Currently, when performing a literature search, there has been no cases found that documents a poorly differentiated cSCC with a synchronous adenocarcinoma of the lacrimal gland. This is mainly because there are improved facilities and accessible healthcare. This report will mainly focus on the presentation and management of this patient case.

**Conclusion:**

To our knowledge this is the first case report documenting a poorly differentiated cSCC and a synchronous adenocarcinoma of the lacrimal gland. Our case report raises the awareness of the possibility of patients with synchronous tumours and is a valuable addition to the literature.

## Highlights

• 47-year old male patient referred for cutaneous squamous cell carcinoma (cSCC) of right upper eyelid.
• Treated surgically by wide local excision and orbital exenteration.
• Histological analysis found an incidental synchronous lacrimal gland adenocarcinoma.
• To our knowledge this is the first case report to describe this phenomenon.

## 1 Background

Cutaneous squamous cell carcinoma (cSCC) is an invasive malignancy with an incidence of 207.5 per 100,000 men per year and 128.8 per 100,000 women per year [1]. Adenocarcinoma of the lacrimal gland has an incidence of less than one case per million per year [2]. We present a patient who experienced a cSCC and adenocarcinoma of the lacrimal gland, which is unusual for both these cancers to present in the same patient in such close proximity. This work has been reported in line with the Surgical Case Report Guidelines (SCARE) criteria [3].

This patient received healthcare in a community hospital.

## 2 Case presentation

The patient signed the consent form, therefore written informed consent to participate in this report has been obtained.

**Fig. 1:**
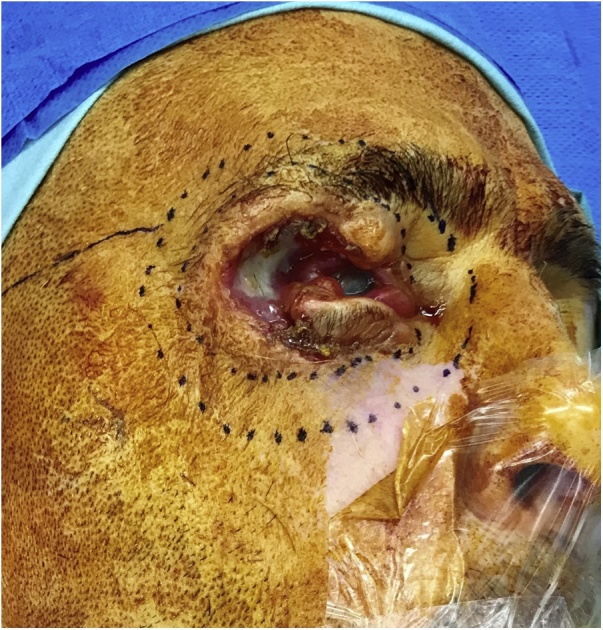
Photograph of the right periorbital area showing extensive invasion of tumour.

A 47-year-old man was referred for management of a cSCC on the right upper eyelid (Fig. 1). The patient had an 8-year history of a right upper eyelid lesion, which was diagnosed as a cSCC on biopsy. He underwent wide local excision with clear resection margins and reconstruction with Cutler-Beard flap in 2018 under ophthalmology. There was no other significant family and past medical history. The patient had a previous history of intravenous drug use but was not under any regular medications or treatments.

Division of the flap was planned but due to patient related factors he was lost to follow up. He was referred to maxillofacial and when he re-attended for division of the flap, he was found to have a new lesion in the lateral aspect of the right upper eyelid and was diagnosed as a recurrent cSCC on biopsy. The patient was subsequently referred to Oral and Maxillofacial Surgery via ophthalmology for further management.

Clinical examination revealed a large 4 × 3.5 cm ulcerated mass in the right lateral periorbital region. The mass compressed the globe and extended from supraorbital ridge to the level of the zygomatic arch. There was also partial loss of vision from the right eye. There was no evidence of cervical lymphadenopathy. A repeat biopsy of the region confirmed poorly differentiated cSCC occupying the entire width and depth (8 mm) of the biopsy specimen.

Staging scans identified no bony erosion or intracranial extension, but there was involvement of the lateral rectus muscle.

Following an multidisciplinary team discussion, he was managed surgically with wide local excision of the tumour with 1 cm margin with an orbital exenteration, a right superficial parotidectomy and reconstruction by means of placement of orbital implants, an anterolateral thigh (ALT) flap [4] and a partial thickness skin graft. A consultant surgeon was the main operating surgeon for this case.

**Fig. 2:**
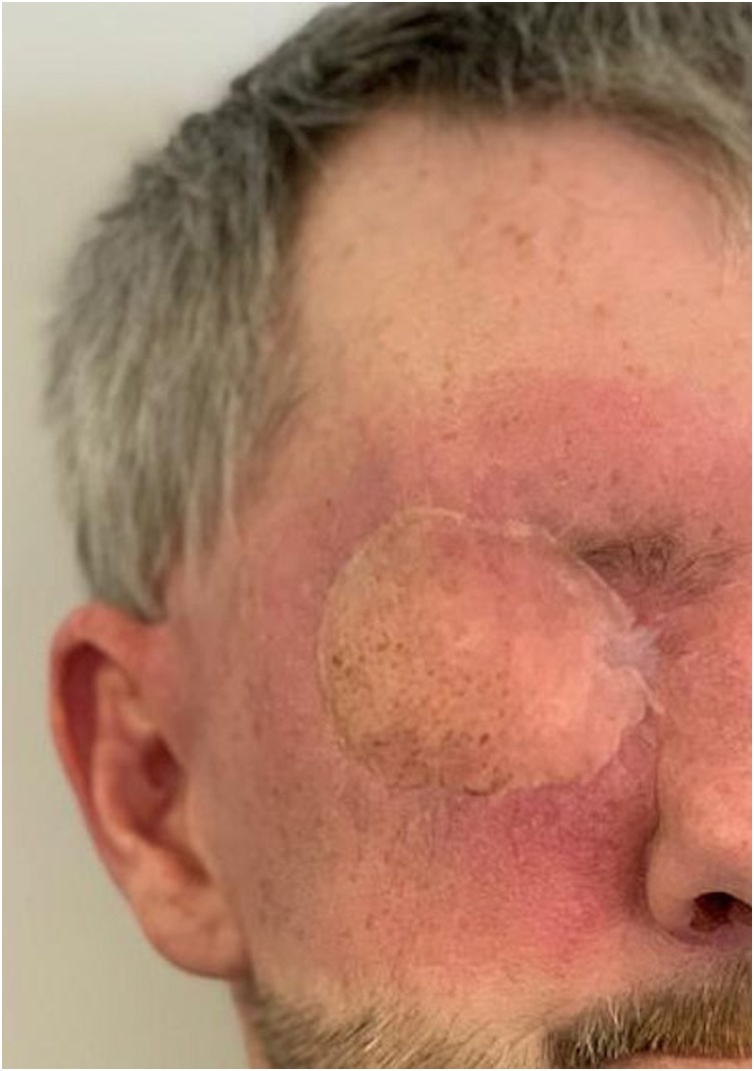
Post-operative photograph of patient at 2 months.

The patient received post-operative care at the community hospital and made good recovery. He has healed without complications as shown in Fig. 2.

**Fig. 3:**
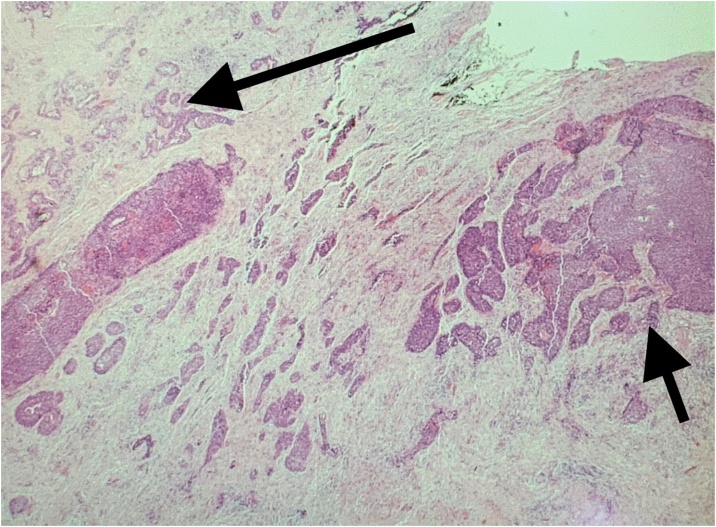
The histopathology slide with the adenocarcinoma labelled using the long arrow and the SCC by the short arrow.

The post-operative histopathological results showed a pT4b, 35 mm × 12 mm SCC with perineural invasion involving bone with tumour present at the deep margin [5]. Additionally, the histology revealed an incidental synchronous pT1a, 20 mm adenocarcinoma of the lacrimal gland with extra glandular extension and perineural invasion focally present at deep margin. The two tumours within the same histology specimen are shown in Fig. 3.

Further management plan involves post-operative radiotherapy and further reconstruction with an ocular prosthesis.

## 3 Discussion

Katz and colleagues were the first to characterise an adenocarcinoma in 1996, which is a type of lacrimal gland tumour [6]. Lacrimal gland tumours itself are very uncommon as this contribute to fewer than 5% of all biopsied orbital lesions [7]. Only 5–7% of epithelial tumours of the lacrimal gland are adenocarcinomas, making them extremely rare [6].

Adenocarcinomas often begin its growth in the upper eyelid and will experience symptoms similar to our patient. Symptoms can include dystopia, exophthalmos, pain and, like in our patient, reduced visual acuity. However, it is common for this tumour to be symptomless, therefore it is more common for patients to present during the advanced stages. It is important for early detection and treatment because adenocarcinomas are very aggressive and invasive [8]. Consequently, the tumour will metastasise early with common sites of metastasis being the liver, brain and lungs [6].

Clinical diagnosis of lacrimal gland tumours is based on symptoms, signs, computed tomography (CT) and magnetic resonance imaging (MRI) scans. From imaging, lacrimal gland tumours have an irregular outline, however it is very common for these tumours to be misdiagnosed as benign.

Currently, the standard treatment is mainly surgical excision with radiotherapy. It is recommended for adenocarcinomas to be completely excised and followed by adjuvant radiotherapy.

SCC is the most common secondary epithelial neoplasms of the orbit region [9]. Subsequently, many are diagnosed early and treated with surgical excision. Similarly, with adenocarcinomas, it is very important for early diagnosis as SCCs are also very invasive and grow rapidly. Thus, when the patient does not get treated quickly, SCCs can cause orbital invasion. Delayed treatment can be caused by a delay in presentation, like in our patient. When there is orbital invasion, symptoms such as pain, haemorrhage and eyelid function abnormalities may be experienced. Orbital exenteration is performed to treat patients, like in our case, who present during this later stage [10].

It is more common for patients who live in rural areas, and hence has poor access to healthcare, or come from low socioeconomic levels to present during the advanced stages. Reasons include the lack of public education on the advantages of early treatment and the lack of health consciousness. As SCC can quickly metastasise, it is important for patients to present early, therefore public education should be improved. The primary site of metastasis are the regional lymph nodes [11].

Currently, when performing a literature search, there has been no cases found that documents a poorly differentiated cSCC with a synchronous adenocarcinoma of the lacrimal gland. This is mainly because there are improved facilities and accessible healthcare. Therefore, tumours are usually treated earlier, unlike our patient who has had the SCC for eight years before presenting to our clinic. Furthermore, given how uncommon adenocarcinomas are, it is very unlikely for this type of tumour to occur with a cSCC in the same orbital region.

## 4 Conclusion

To our knowledge this is the first case report documenting a poorly differentiated cSCC and a synchronous adenocarcinoma of the lacrimal gland. Our case report raises the awareness of the possibility of patients with synchronous tumours and is a valuable addition to the literature.

## Funding

No sources of funding.

## Ethical approval

This study was exempted from ethical approval.

## Consent

Consent form has been signed by the patient for approval.

## Author contribution

CH posed the idea of producing a case report from the interesting case and wrote the manuscript and case report. KS edited the manuscript for the case report. BV acquired the necessary photos, clinical notes and letters from the patient. MD gathered the histopathology photo and results. All authors approved the final draft of the manuscript before submission.

## Registration of research studies

N/A.

## Guarantor

Carmen Lok Tung Ho.

## Provenance and peer review

Not commissioned, externally peer-reviewed.

## CRediT authorship contribution statement

**Carmen Lok Tung Ho:** Writing - original draft, Writing - review & editing, Conceptualization. **Bhavin Visavadia:** Writing - review & editing, Investigation. **Keval Shah:** Writing - original draft, Writing - review & editing. **Manjiri Deshmukh:** Writing - review & editing, Investigation.

## Declaration of Competing Interest

No conflicts of interest.
